# Cognitive-Motivational Determinants of Residents’ Civic Engagement and Health (Inequities) in the Context of Noise Action Planning: A Conceptual Model

**DOI:** 10.3390/ijerph14060578

**Published:** 2017-05-30

**Authors:** Natalie Riedel, Irene van Kamp, Heike Köckler, Joachim Scheiner, Adrian Loerbroks, Thomas Claßen, Gabriele Bolte

**Affiliations:** 1 University of Bremen, Institute of Public Health and Nursing Research, Department of Social Epidemiology, Grazer Straße 4, 28359 Bremen, Germany; gabriele.bolte@uni-bremen.de; 2 Centre for Sustainability, Environment and Health, National Institute for Public Health and the Environment RIVM, Antonie van Leeuwenhoeklaan 9, 3721 MA Bilthoven, The Netherlands; irene.van.kamp@rivm.nl; 3 Hochschule für Gesundheit (University of Applied Science), Department of Community Health, Gesundheitscampus 6-8, 44801 Bochum, Germany; heike.koeckler@hs-gesundheit.de; 4 TU Dortmund University, Faculty of Spatial Planning, Department of Transport Planning, August-Schmidt-Str. 10, 44221 Dortmund, Germany; joachim.scheiner@tu-dortmund.de; 5 University of Düsseldorf, Faculty of Medicine, Centre for Health and Society, Institute for Occupational, Social, and Environmental Medicine, Universitätsstraße 1, 40225 Düsseldorf, Germany; adrian.loerbroks@uni-duesseldorf.de; 6 Centre for Health NRW (North Rhine Westphalia), Section “Health Assessments and Forecasting”, Westerfeldstr. 35/37, 33611 Bielefeld, Germany; thomas.classen@lzg.nrw.de

**Keywords:** residential traffic noise exposure, noise annoyance, behavioural outcome expectancy, perceived behavioural control, coping, civic engagement, health inequities, environmental justice, logic model

## Abstract

The Environmental Noise Directive expects residents to be actively involved in localising and selecting noise abatement interventions during the noise action planning process. Its intervention impact is meant to be homogeneous across population groups. Against the background of social heterogeneity and environmental disparities, however, the impact of noise action planning on exposure to traffic-related noise and its health effects is unlikely to follow homogenous distributions. Until now, there has been no study evaluating the impact of noise action measures on the social distribution of traffic-related noise exposure and health outcomes. We develop a conceptual (logic) model on cognitive-motivational determinants of residents’ civic engagement and health (inequities) by integrating arguments from the Model on household’s Vulnerability to the local Environment, the learned helplessness model in environmental psychology, the Cognitive Activation Theory of Stress, and the reserve capacity model. Specifically, we derive four hypothetical patterns of cognitive-motivational determinants yielding different levels of sustained physiological activation and expectancies of civic engagement. These patterns may help us understand why health inequities arise in the context of noise action planning and learn how to transform noise action planning into an instrument conducive to health equity. While building on existing frameworks, our conceptual model will be tested empirically in the next stage of our research process.

## 1. Introduction

In response to the substantial exposure to traffic-related noise in urban areas [1], the EU Environmental Noise Directive (END, 2002/49/EC) requires public environmental authorities to map environmental noise and to implement noise action plans for noise abatement [2]. With its universal objective to protect and improve European populations’ health, noise action planning implicitly complies with assumptions underlying population-based prevention strategies [3]. This prevention approach relies on the average: Average exposure and response values apply to the majority of the population and, consequently, the intervention impact is expected to be relatively homogeneous across population groups. Against the background of social heterogeneity and environmental disparities in urban societies [4,5,6,7], however, the intervention impact of noise action planning on exposure to traffic-related noise and its health effects is rather unlikely to follow homogenous distributions.

Noise action planning is bound to be highly selective in terms of the contents, place, and timing of actions. Only recently, the application of different noise priority indices used in the context of noise action planning has been shown to produce spatially divergent outcomes for noise actions [8]. Fifteen years after the implementation of the END, the third round of noise mapping and action planning is lying ahead, but there has been no study evaluating the impact of noise action measures on the distribution of traffic-related noise exposures and health outcomes among population groups.

Evidence on (unintended) intervention-generated inequalities in exposures or health outcomes in the field of environmental health is scarce and interventions dealing with the management of traffic flows have yielded mixed results. Social inequalities in road causalities were still maintained in one study, because the intervention failed to rule out underlying causes for spatial patterns [9]. In another study, social inequalities in air quality and noise pollution increased, as the greatest benefit was observed in higher status areas [10]. By contrast, a congestion charge diminished the socio-spatial differences in air pollution and life expectancy to some extent [11]. This study investigated the social differences in exposure, but not in vulnerabilities relevant for social inequalities in the health effects of environmental exposures. Thus, interventions like noise action planning need to consider changes in social distributions of exposure and health-related responses following noise abatement measures (distributional environmental justice).

Such considerations start by reflecting procedural features of the policy cycle (procedural environmental justice). In the case of noise action planning, transparent environmental health information and effective participation of the public are ingredients of the policy process. They are decisive for its outcome, since there is no binding standard for noise action values and noise action planning is subject to the agency of those motivated to participate in the planning process [2,12].

It is difficult for non-experts to comprehend the contents of noise maps and to draw adequate conclusions for specific local exposure situations [13,14]. Pilot projects in Germany and Italy impressively demonstrated what comprehensive ‘information and consultation’ according to the END might entail (e.g., performing surveys and soundwalks in selected areas, targeting population groups using places for different purposes, presenting survey results to residents, organising workshops with residents, mediating between parties including polluters, supporting residents to communicate their suggestions, and creating new ‘soundscape-based layouts’ of places, etc. [15,16]). However, these approaches assume pre-existing interest, motivation, and readiness for civic engagement. As was reported for the German pilot project, fewer residents took part in the participation activities than expected. Instead of direct communication between local environmental agencies and residents, written communication methods are prevailing in Germany [17], with municipalities increasingly using the internet (so-called e-participation). E-participation can reach relatively high response rates, as documented for a German city after large public campaigns [18]. However, the response is highly selective. Among registered users, two thirds of participants had attained at least a university entrance level, were male, and were between 30 and 60 years old, which is a well-known pattern of civic participation in urban environmental planning [19].

Findings of low and selective responses to participation opportunities are not surprising from both psychological and epidemiological viewpoints: psychologically, unequal readiness for civic engagement in noise action planning may result from cognitive distortions. Agreements with statements like “the effects of noise are overestimated” were more common among lower educated participants in an Austrian study [20]. Social epidemiological reasoning further suggests that intervention strategies building on individuals’ motivation and capacity to participate may magnify health inequities [21]. Research in the field of environmental justice contends that a limited individual coping repertoire explains socio-political vulnerability among residents in lower social positions [22,23,24]. Neglecting structural constraints and different vulnerabilities, noise action planning—like any other planning instrument—expects residents to engage as active citizens.

In light of the END, it is of utmost importance to gain further insights into the determinants of residents’ motivation and capacity to take interest in the environmental quality of their residential environment and to be ready for civic engagement in END noise action planning. For this reason, we aim to develop a conceptual model on the cognitive-motivational determinants of residents’ civic engagement and health (inequities) in the face of chronic traffic noise exposure. This model is intended to reveal why health inequities and equity impacts should be considered by noise action planning and where planners could intervene to mitigate, prevent, or even reverse the generation of inequalities. Thus, the outcome of this conceptual article can be regarded as a logic model presenting possibly causal (inter-)relationships between contextual and individual determinants relevant for intervention planning and evaluations [25,26]. At this stage, our model is composed of elements from existing frameworks that have previously been subject to empirical studies. Its empirical validation as a whole is planned in the next stage of our research process.

The components of our model and their conceptual links are developed as follows: We take the “Model on household’s Vulnerability to the local Environment” (MOVE) [27,28] as a valuable point of departure, because it presents predictors of “institutional coping behaviour” in environmental matters (Section 2). It is the first model (in Germany) dealing with environmental planning that takes environmental psychology into account and is framed by environmental justice. Further insights from environmental psychology concerning noise-induced helplessness (Section 3.1) allow us reframe MOVE based on the psychobiological Cognitive Activation Theory of Stress [29,30] (Section 3.2) as a first step towards our refined conceptual model (Section 4). In a second step, we align our arguments with the reserve capacity model that focuses on resource aggregates and emotions as determinants of health inequalities [31,32,33], thereby bridging psychology and epidemiology. In reference to MOVE and the reserve capacity model, we further add psychosocial and tangible resources involved in unequal health and participation chances (Section 5). By integrating these components, we present a new conceptual model explaining both the unequal effects of noise exposure and unequal participation simultaneously.

## 2. Model on Household’s Vulnerability to the Local Environment (MOVE)

Positioned at the interface between health and environmental psychology, as well as environmental urban planning with a strong behavioural emphasis, MOVE [27,28] was conceived to explain residents’ intention and self-reported behaviour of civil actions, e.g., collecting signatures, joining or founding a local initiative. Thus, MOVE deals with residents’ institutional coping as an active, problem-focused response to air and noise pollution. Based on the Theory of Planned Behaviour (TPB) [34,35,36], MOVE describes cognitive-motivational and actual predictors of institutional coping that reflect positive expectancies of coping outcomes (as captured by behavioural attitude and subjective norm) and of coping-specific perceived controllability and self-efficacy (as an important aspect of perceived behavioural control). These expectancies arise from three beliefs:

1. Behavioural beliefs (“the subjective probability that the behaviour will produce a given outcome” [37]) engender a specific *attitude towards institutional coping behaviour*. In MOVE, a resident is more likely to choose an institutional coping strategy if she or he expects her or his engagement with environmental quality to be worthwhile (perceived value of the outcome of institutional coping behaviour), prefers quietness, and rates environmental quality as low (as measured by annoyance).
2. Normative beliefs (“the perceived behavioural expectations of [..] important referent individuals” [37]) bring about residents’ *subjective norm* and social pressure to initiate MOVE institutional coping. A resident’s inclination to institutional coping behaviour is more pronounced if she or he anticipates positive reactions from those who are close and important to her or him. In a way, the subjective norm also represents positive outcome expectancy.
3. Control beliefs (“perceived presence of factors that may facilitate or impede performance of a behaviour” [37]) become manifest in residents’ *perceived behavioural control*. Ajzen [35] distinguishes two aspects of perceived behavioural control: behavioural controllability (“belief about the extent to which performing behaviour is up to the actor”) and self-efficacy (“ease or difficulty of performing a behaviour”). The more a resident assumes to know about objective environmental quality and environmental rights, the greater her or his engagement-specific controllability. Equally, the more she or he feels confident and capable of civic engagement, the more self-efficacy regarding civic engagement is present. The stronger engagement-specific controllability and self-efficacy are, the higher the chance that a resident will adopt an institutional coping strategy.

While attitude, subjective norm, and perceived behavioural control correlate, each can be seen to contribute to explaining behavioural intentions as predictors of self-reported (performed) coping behaviour. Moreover, the perceived behavioural control indicates the objective, factual behavioural control and can determine the self-reported coping behaviour directly.

However, MOVE does not solely build on the TPB. MOVE elaborates on the actual behavioural control that Ajzen is aware of in his theory, but it has never been explored or operationalised, or even tested empirically. MOVE therefore complements the TPB with the Conservation of Resources (COR) Theory [38,39], an established stress theory in health psychology. In brief, COR theory purports that stress results from the feared and suffered loss of the resources that an individual needs to maintain for her or his well-being. An individual is at an elevated risk of further resource loss if she or he fails to prevent resource loss or to (re-)gain adequate resources after resource investment. Such dynamics of increasing vulnerability are portrayed as “loss cycles”, which corresponds to the observation that “resources tend to occur in aggregate [giving evidence of successful resource gain or gain cycle, added by authors], or be absent in the aggregate” [31], p. 147 (in reference to Hobfoll, see the reserve capacity model in Section 5). Conversely, success in resource maintenance may stop the loss cycle and enable an individual to gain resources. If perceived behavioural control is a major determinant of actual institutional coping, MOVE provides a compelling explanation for loss cycles related to environmental conditions (if they are perceived to be a threat to health and well-being). In emphasising the role of resources for the experience of stress, COR theory acknowledges the objective, social, and physical setting of the psychological stress mechanism.

Whilst studied as a typical outcome of stress-related processes in epidemiology and psychology, COR theory regards (environmental) health as a resource vital to an individual’s development and agency. Consequently, perceived behavioural control depends on the availability and conversation of resources. Based on COR theory [38], MOVE outlines four broad classes of resources essential for stress resilience, perceived behavioural control, and self-reported institutional coping behaviour:

- “Objects”: They are appreciated because of their “physical nature” and/or the status that individuals attribute to their acquisition or ownership (e.g., home ownership).
- “Personal characteristics”: They are supportive of a “positive sense of self and a view that one can master or at least see through stressful circumstances”. An example of personal characteristics is the communal mastery, which is an individual’s belief to be able to reach goals or to overcome challenging situations due to being affiliated with others [40]. MOVE specifies communal mastery by residents’ perceived collective competence to exert control over their home and neighbourhood. Communal mastery is theoretically related to the aspect of self-efficacy integrated in the perceived behavioural control of institutional coping, but expanded by a pro-social aspect of collective action.
- “Conditions”: They define either personal circumstance (e.g., age) or are desired for their own value and for being a key to other resources (e.g., social networks).
- “Energies”: They provide the means to gain resources in the previous categories (e.g., income, knowledge).

We are aware of the conceptual overlap between Hobfoll’s personal characteristics and Ajzen’s behavioural, normative, and control beliefs, which supports the MOVE model’s reasoning to combine both theories. The same may apply to knowledge as an energy resource that MOVE considers as inherent to behavioural control expectancies.

Considering that noise action planning requires positive expectancies of institutional coping, MOVE appears to be a useful conceptual basis to study participation chances in environmental noise policies. In the first empirical test of MOVE [27], most study participants reported no institutional coping behaviour. This observation was mainly explained by a lack of confidence. To learn more about those who do not engage in institutional coping despite being exposed to environmental burdens was one of the questions raised for further research. The empirical test did not confirm that objective noise exposure is a determinant of institutional coping behaviour. Yet, self-reported institutional coping behaviour varied by exposure levels in bivariate analyses. To increase the explanatory value of the model, additional factors need to be identified. This is why our adoption of MOVE may benefit from an integration of research on the cognitive-motivational impacts of the environmental exposures that residents are expected to actively cope with.

## 3. Integration of Noise-Induced Helplessness and Cognitive Activation Theory of Stress

For our purpose, we will briefly describe the environmental psychological observations of learning helplessness under conditions of chronic noise exposure (Section 3.1) whose general impact on residents’ health, as well as their motivation and capacity to act as active citizens, is theorised from a cognitive, learning viewpoint (Section 3.2).

### 3.1. Noise-Induced Learned Helplessness vs. Civic Engagement for Health?

Instead of active engagement, environmental psychological research referring to the learned helplessness model rather suggests a higher probability of passivity and social withdrawal in response to unavoidable, uncontrollable, and adverse environmental exposures like air and noise pollution (cf. [41] for the first laboratory study on learned helplessness in humans, cf. [42,43,44,45] for reviews on the application of ‘learned helplessness’ in the field of environmental psychology). Exposure to learned helplessness in a given situation may generalise to other situations, even if completely different competences are required [46]. According to the reformulated learned helplessness model [47,48,49,50], the severity of helplessness and its health impact depend on an individual’s characterization of its cause (“causal attribution”) and the affective value of the uncontrollability experience (i.e., the personal importance of the feared or desired event).

Traffic-related noise has been described as an ambient or environmental stressor that is chronic and intractable [51]. If intractability implies that traffic-related noise cannot be removed by any individual’s “isolated effort” ([51] p. 363) and will not cease due to its chronicity, residents may learn they cannot exert any control over their environment and that the same applies to all those who are relevant, such as close neighbours. The unpredictability of noise peaks may further contribute to learned helplessness [42]. Such a characterisation of uncontrollability may result in universal, communal helplessness shared by noise-exposed residents (see additionally [47] pp. 52–53), which, in turn, may hamper interest in community issues and in socio- and eco-political engagement. In addition, communication disturbance, withdrawal from the environment outside the dwelling, diminished place attachment, and a lack of social support in the neighbourhood have been described as “social costs” of high traffic loads in the residential environment [52,53,54]. The affective value of perceived uncontrollable noise exposure may moderate the psychological stress reaction, as expressed by noise annoyance [55,56].

Surprisingly, environmental psychological research has paid little attention to cognitive-motivational determinants of residents’ capacity to claim rights for environmental health in the community context, despite recognizing the relevance of perceived control over noise exposure and coping strategies for noise annoyance, well-being, and health [55,57,58,59,60], mainly in reference to Lazarus’ appraisal theory of stress or its adoption by Stallen [55]. Instead, residents’ distrust in regulatory authorities has been considered as a determinant of controllability perceptions and the coping capacity preceding noise annoyance [55,59], or as one psychological determinant of noise annoyance among others [61,62,63]. Changes to task performance and persistence, such as the number of attempts until withdrawal from unsolvable tasks or the time until an effective solution is presented after suffering failure, have been employed by previous studies on learned helplessness [45].

Therefore, we need to understand how noise-specific helplessness may spill over to affect residents’ expectancies of MOVE institutional coping and health. We introduce a cognitive learning perspective, as developed by the Cognitive Activation Theory of Stress, that may improve our understanding of the relationship between cognitive-motivational determinants and residents’ participation and health chances.

### 3.2. The Cognitive Activation Theory of Stress (CATS)

The Cognitive Activation Theory of Stress (CATS) describes the stress process and its health sequelae from a psychobiological perspective [29,30]. It provides additional theoretical underpinnings of early findings for the learned helplessness model. CATS has been theorised and examined with regard to stressful work environments [64,65,66], but it has also been referred to as a stress model explaining the effects of noise as an ambient stressor on annoyance and health ([56] pp. 153–154, [57] using a predecessor of CATS combined with Lazarus’ stress model).

According to CATS, the stress response originates from a perceived “discrepancy between what is expected to be ‘normal’ (set value [in CATS equated with goals, added by authors]) and what is happening in reality (actual value)” [29]. This perceived discrepancy (i.e., the stressor) is assumed to induce neurophysiological activation. Referring to COR theory and an environmental psychological notion of noise annoyance [55], we propose that this discrepancy results from the perceived uncontrollability of noise exposure eroding residents’ capacity to maintain resources, including their health.

Neurophysiological activation due to this discrepancy remains elevated until the organism has succeeded in reducing the disparity between expected (feared or desired) and actual values (of personal noise exposure, for example). If the difference between expected and actual values persists, arousal sustains and induces pathophysiological processes. Such feedback of the stress response to the brain has been termed the “allostatic load” (i.e., adaptive costs, physiological strain) elsewhere [67], though it was mainly explained by repeated exposures to stress stimuli and subsequent over-stimulation. In CATS, however, “the real concern is sustained arousal occurring when there is no solution” ([30] p. 879), as the organism is vainly struggling for homeostasis by trying to exert control on expected and/or actual values. This postulation appears to be in agreement with the earlier discussed notion of learning helplessness in response to uncontrollable traffic noise exposure (see Section 3.1).

The higher the individual rates her or his chances to reduce or remove the difference between expected and actual values, the more likely it is for the organism to return to the arousal level observed prior to the activation. At this point, CATS introduces two types of expectancies essential for the cognitive evaluation of the incongruity between expected and actual values: stimulus and outcome expectancies. In accordance with the learned helplessness model, stimulus and outcome expectancies are learned information about stimulus-stimulus relations and behavioural response-outcome (non-)contingencies.

The stimulus expectancy arises from the perceived probability of an event associated with a specific stimulus, such as sleep disturbance following peaks of traffic noise. The degree of “predictability” is mentioned as the critical feature of stimulus expectancies, which is all the more important for traffic noise (see section above) [42]. It is by changing the perceived probabilities based on the subjective stimulus characterisation and/or affective values that an individual may defend herself/himself against a prevailing discrepancy between expected and actual exposure values. As exposed residents do not perceive immediate harm (termed “non-urgency” [51]), they may be inclined to accept the presence of the environmental stressor and cling to distracting cognitions in order to reduce the cognitive dissonance. A statement like “Living in a big city I have to put up with the traffic noise in my home” indicates disengagement and is called stimulus distortion. Noise annoyance decreases in this instance, even though there is no physiological habituation to chronic noise exposure.

Outcome expectancies are closely linked to the stimulus expectancies and refer to the perceived relationship between actions (behaviours) and their results (outcome). CATS postulates three types of outcome expectancies: positive effects, no control, and negative effects.

Positive outcome expectancy is practically equated with active coping. This type of outcome expectancy is comparable in nature to the TPB predictors of MOVE, representing positive specific expectancies of behavioural outcomes (attitude and subjective norm) and behavioural control (especially self-efficacy). However, CATS outcome expectancies are located on a more general level.

An expectancy of having no control implies helplessness. At least in the first stage, helplessness is associated with physiological activation. If the uncontrollability of personal exposure is perceived as a constant or increasing threat to resource maintenance and goal attainment [55] (termed motivational salience [51]), the affective value may become critical and further contributes to the stress experience and noise annoyance. Even if residents are able to reduce their individual traffic noise exposure by staying in their homes, they may feel constrained because of the involuntary retreat at the expense of restoration [68] or the involuntary residential location at a highly trafficked street [51] (see the discussion on residential segregation as “determinant of environmental inequalities” [24]). On the other hand, arousal levels are suggested to decrease if an “individual ‘accepts’ that there is no solution […]. Arousal may also be reduced if the helplessness leads to secondary gain and support from society. In such cases, helplessness may function as a coping strategy, and the secondary gain may reinforce and sustain the helplessness condition” [29] (p. 578). If this assumption holds true, generalised helplessness will not be fatal by itself, but through continued adverse exposures, which brings us back to the link between distributional and procedural environmental justice (see our introduction, Section 1, and [69]).

Negative outcome expectancy implies hopelessness. Generalised hopelessness produces a sustained high arousal, because the individual reproaches himself/herself for failure, in spite of having control over his/her life. Reduced self-esteem, depression, and social withdrawal and isolation may follow from a generalised negative outcome expectancy, all being counteractive to residents’ active agency in the political context, as implied by MOVE [23,27,28].

Bivariate results from the first MOVE field study on roughly 300 residents in a densely populated area in Germany might provide some indication of noise-induced passivity. However, it is important to note that we have to treat these observations with caution, as residents’ social positions were not considered as a confounding variable [28]. A positive outcome expectancy related to the perceived value of civic engagement (i.e., aspect of the attitude towards institutional coping), as well as confidence and knowledge (i.e., behaviour-specific self-efficacy and controllability as the two aspects of perceived behavioural control), were found to be significantly lower among residents in more exposed residential areas as compared to their less exposed counterparts. More exposed residents had lower values on the communal mastery scale. Thinking of “universal helplessness”—indicating that residents project their own helplessness to other significant people (see Section 3.1 above)—it is remarkable that the subjective norm was somewhat less evident under conditions of higher environmental exposures. However, this difference was not statistically significant. Within the CATS frame, “it is the perceived relationship that counts for stress responses and stress consequences, not the objectively true contingencies” ([29] pp. 574–575). Thus, the degree of perceived uncontrollability turned out to be more important for reading and sleeping impairments and psychophysiological symptoms than the objective noise exposure, for example [58]. The perceived uncontrollability of environmental exposure was not accounted for in the MOVE model, which might have led to an underestimation of the true differences in cognitive-motivational determinants between levels of environmental pollution. On average, the choice of coping strategies (problem-focused incl. institutional coping, avoidance, and comforting cognitions) did not vary by objective noise levels and perceived control over noise exposure in a study that was conducted almost 30 years ago [57]. However, perceived control over noise exposure significantly strengthened the association between stress due to noise, and avoiding and comforting coping strategies (avoidance under conditions of air traffic exposure and comforting cognitions under conditions of road traffic exposure, see note 1 at the end of this paper).

## 4. First Step towards a Refined Model: Reframing MOVE Based on CATS

MOVE is a suitable starting point to explore the cognitive-motivational determinants of civic engagement in response to residential exposure to noise and air pollution. However, the MOVE study could not explain the missing link between exposures to environmental burdens and institutional coping behaviour (see the end of Section 2). We therefore outline our first step towards a refined model by delivering general considerations on model refinements (Section 4.1) and proposing hypothetical patterns of cognitive-motivational determinants as model specification (Section 4.2).

### 4.1. General Considerations: Reframing MOVE Based on CATS

Unlike MOVE, we mainly regard noise annoyance as a psychological stress response to perceived uncontrollable noise exposure related to (sustained) physiological activation, thereby complying with the significance that the END attributes to noise annoyance as a focal health indicator. Instead of being an integral part of the attitude, noise annoyance is an intermediate health outcome modulated by cognitive-motivational processes. These processes are initiated by the (un-)controllability experience induced by chronic noise exposure and characterised by a specific stimulus expectancy (belief that the situation of (un-)controllability is permanent or transient) and specific outcome expectancy (perceived (in-)capability to find a bearable solution concerning the personal noise exposure indoors). By integrating both types of expectancies in our conception of the noise-related stress stimulus variable, we acknowledge the interdependence between the two at any point in the cognitive process. The stress effect of (un-)controllability is modified by the affective value that residents assign to their noise exposure, i.e., the extent to which they (would) find it important to be able to reduce the noise exposure in their dwelling. This means that the affective value indicates the degree of discrepancy between the expected and set values in CATS terminology. Further, the expectancies of the stimulus of “(un-)controllable personal traffic noise exposure”, of what can be done about it (perceived behavioural control), and of what the result will be (outcome expectancies as specified by the attitude and subjective norm) are shaped by and re-shape the generalised outcome and stimulus expectancies during one’s lifetime.

CATS does not make clear distinctions between levels of generalization concerning stress stimuli. We propose two additional, higher-order stimulus expectancies affecting residents’ perceptions and appraisal of life (including their noise situation). These are noise sensitivity, conceived to additionally explain or to exacerbate environmental stress reactions, especially noise annoyance (e.g., [42,70,71] for overviews, see [72] for an empirical study by way of an example; see note 2 at the end of this paper), and the negative and positive affect assumed to influence psychobiological processes, including behavioural outcome expectancies, actual coping behaviour, and health (e.g., [73,74,75]). Relationships between generalised stimulus and outcome expectancies are probably reciprocal over time. While we hypothesise positive associations between negative affectivity and no control/negative expectancies, and between positive affectivity and positive outcome expectancy, we acknowledge the co-existence of both emotional states (negative and positive affect). Affectivity and cognitive adaption to the noise stimulus are closely related: Changing the affective value to close the gap between the desired and perceived control over traffic nose exposure is probably a way of cognitive distortion related to negative affectivity (defence response according to CATS).

The environmental psychological interpretation of the learned helplessness model and CATS shed light on the outcome expectancies as triggers of loss and gain cycles according to Hobfoll. The more experiences contributing to the feeling of environmental uncontrollability (or controllability), the more negative (or positive) future expectancies of behavioural outcomes and control as expressed by TPB attitude, subjective norm, and perceived behavioural control. We add communal mastery to these specific positive outcome expectancies, given its conceptual relatedness to the aspect of self-efficacy inherent to TPB perceived behavioural control and the observation of social withdrawal as a helplessness symptom. In turn, attitude, subjective norm, perceived behavioural control, and communal mastery are positively associated with residents’ intentions to be engaged in institutional coping. In addition, both perceived behavioural control and communal mastery will directly enhance the chance of actually performed institutional coping behaviour. If effective, institutional coping behaviour diminishes noise annoyance. On the other hand, the defence response (disengagement) is inversely related to the MOVE positive outcome expectancies and noise annoyance.

Our conceptual model highlights the cognitive-motivational processes related to the ambient stressor of ‘traffic-related noise’ (indirect pathway to health). Nonetheless, it is the objective traffic-related noise exposure that requires planners to combat traffic-related noise and causes direct pathophysiological changes (cf. [76,77] for reviews recognising both pathways and see the “noise reaction model” in [78,79]). The objective intractable presence of traffic-related noise in residential areas is still the impetus for the cognitive-motivational and psychobiological processes related to perceived uncontrollability, as implied by the differences in selected TPB predictors and self-reported coping behaviour between exposure levels in the MOVE study (see the end of Section 2 and Section 3.2). The conceptual model focuses on generalised expectancies of stimuli (i.e., positive and negative affect) and behavioural outcomes (positive, no control, or negative), as well as noise annoyance as bio-psycho-social links between cognitive-motivational and physiological changes.

**Figure 1:**
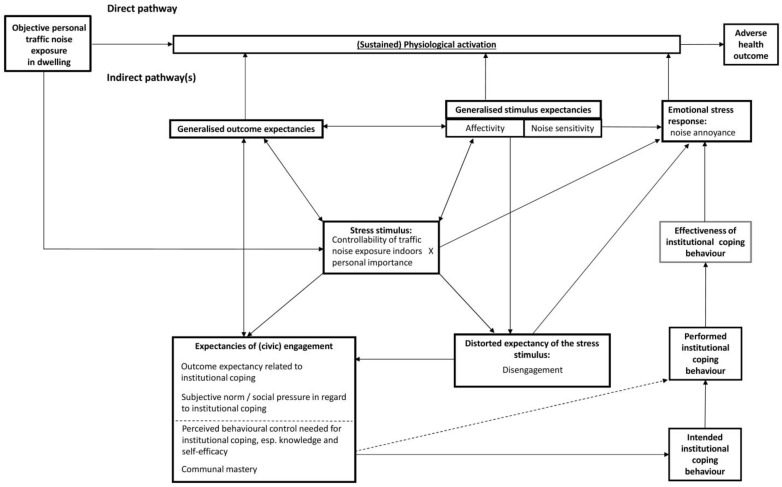
First step towards a refined model: reframing MOVE based on CATS.

Our general considerations are depicted in the conceptual model in Figure 1.

### 4.2. Specification of Our Model: Hypothetical Patterns of Cognitive-Motivational Determinants

In the frame of our model, we may investigate four constellations characterised by different levels of noise annoyance and specific hypothetical patterns of cognitive-motivational determinants (see Table 1):

1. perceived high controllability of traffic noise exposure indoors x low personal importance (affective value),
2. perceived high controllability of traffic noise exposure indoors x high personal importance,
3. perceived low controllability of traffic noise exposure indoors x low personal importance, and
4. perceived low controllability of traffic noise exposure indoors x high personal importance.

We expect to detect the lowest levels of noise annoyance in residents reporting low personal importance while perceiving high controllability (group 1). As the expected and actual controllability should largely match among residents from group 2, they could show low levels of annoyance. However, noise sensitivity is probably linked to high personal importance, which may partially explain its modifying effect on noise annoyance. In consequence, noise annoyance is likely to reach moderate levels. In the residents from group 3, levels of annoyance due to low controllability should be reduced by the buffering effect of a low personal importance. The discrepancy between expected and actual controllability is highest among residents from group 4. Stating a high personal importance, while perceiving low controllability and high noise sensitivity, they are suggested to be highly annoyed.

The hypothetical patterns within groups 2 and 3 are considered as stable, whereas divergences between controllability and personal importance within groups 1 and 4 lead us to assume that cognitive changes could occur in the face of changes in living conditions and objective noise exposure. For this reason, there might be opposite patterns within these two groups concerning the inter-relations between generalised and specific expectancies and subsequent physiological activation, as marked by the subgroups A and B in Table 1.

Group 1A: Emotionally unaffected due to high controllability and low personal importance, residents are characterised by a high positive affect and generalised positive outcome expectancy. There is capacity for engagement, but low personal importance hints at preferences or necessities other than high environmental quality. Overall, this leads to moderate (dis-)interest in institutional coping strategies. Compared to other groups, these residents have the lowest level of physiological activation.

Group 1B: Alternatively, low personal importance indicates a high negative affect and generalised helplessness learnt in previous contexts. Residents tend to disengage from their (residential) environment in general. While resignation reduces physiological activation, a negative affect may still sustain a moderate arousal.

Group 2: Residents display a high positive affect and generalised positive outcome expectancy. In consequence, these residents are the most inclined to adhere to positive expectancies of institutional coping. In accordance with noise annoyance and noise sensitivity levels, physiological activation is moderately increased.

Group 3: Low personal importance is indicative of generalised helplessness. Residents show a high negative affect, apply cognitive distortion, and withdraw from their residential environment. Thus, they have low expectancies of institutional coping behaviour. Physiological activation is moderately elevated by a negative affect and noise annoyance.

Group 4A: High personal importance presents evidence of a lasting discrepancy between actual and expected controllability and of feeling guilty of failure, which entails a high negative affect and generalised hopelessness. Residents apply cognitive distortion and refrain from institutional coping. In comparison to the other groups, they suffer from the highest sustained arousal.

Group 4B: High personal importance reflects a positive affect and generalised positive outcome expectancy, pushing residents to stand up against the perceived uncontrollable noise exposure and to get involved in institutional coping. Thanks to strong psychosocial resources, physiological activation remains moderate.

According to these hypothetical patterns, health inequities may particularly arise from: (a) increased vulnerability to environmental pollutants due to sustained arousal in residents from group 1B and 4A; and (b) under-representation of residents from group 3 and 4A as compared to residents from group 2 and 4B in public participation, even if noise exposure levels are comparable among these groups. At last, a lack of awareness among residents from group 1A might be a drawback to noise action planning in situations when environmental health protection has to compete with other interests associated with motorised traffic. In political debates, residents from this group might contribute to health inequities indirectly, by arguing against noise abatement measures that they perceive as mobility constraints.

## 5. Second Step towards a Refined Model: Adding Resources

Unequal access to resources explains differences in self-reported institutional coping behaviour between population groups in MOVE. COR theory defines resources at the intra- and inter-individual level. To embed our model on cognitive-motivational determinants of civic engagement and environmental health (inequities) in social epidemiological research, we additionally present the reserve capacity model developed to examine psychosocial, intra-, and inter-individual factors in the relationship between social positions and physical health outcomes [31,32,33]. We are the first to use its conceptual ideas in the context of environmental health and of distributional and procedural environmental justice.

In this second step, we essentially abide by Hobfoll’s notion of resources (objects, personal characteristics, conditions, and energy resources) as such (see MOVE in Section 2), but re-arrange them as elements of psychosocial resources (incl. personal characteristics and condition resources, Section 5.1) and tangible resources (incl. objects, condition and energy resources, Section 5.2), according to the reserve capacity model and as constituents of residents’ social position (incl. condition and energy resources, Section 5.3).

### 5.1. Psychosocial Resources

In principle, the pathways to health inequalities in the reserve capacity model appear familiar in social epidemiological research interested in bio-psycho-social mechanisms [80,81,82,83] (see also [84] pp. 191–201 for a concise overview, see [85] for a conceptual model in the neighbourhood context): Social differences in chronic or repeated stress experienced in different contexts (e.g., home, work, and neighbourhood environments) cause inequalities in stress reactivity, in stress-related physiological changes, and, finally, in morbidity and mortality (we may refer to this as the “direct pathway”). The notion of stress as a “threat of or actual loss of resources” is borrowed from COR theory. Another pathway emphasises the emotions (e.g., anger, frustration, anxiety, depressive symptoms; we may add noise annoyance here) and cognitions that individuals develop in the stress response. These emotions and cognitions are transformed into pathophysiology and mal-adaptive behaviour as predecessors of mortality and morbidity. The cumulative loss of resources accounts for the enhanced stress reactivity among individuals of lower social positions [31]. Low social positions are associated with contexts posing high demands, while making it difficult to restore and to augment resources. An increasing disparity between the resources spent and resources gained undermines an individual’s reserve capacity (that is her or his resource aggregate). In the emotional-cognitive pathway to health inequalities, the reserve capacity is located between stress as defined by resource loss and cognitive-emotional differentials. A diminished reserve capacity partly mediates or moderates negative cognitions and emotions, mal-adaptive behaviours, and pathophysiological processes.

Empirical research mainly investigated the role of psychosocial resource aggregates (as constructed by composite variables aggregating resources, see note 3 at the end of this paper). When considered with COR theory, we think that the generalised expectancies of stimuli and outcomes and civic engagement are psychosocial resources (personal characteristics in Hobfoll’s COR theory) arising from interactions with the objective environment, as learning processes take place in contexts characterised by quantitatively and qualitatively different resources (and stressors). Accordingly, the learning, cognitive-motivational, and biological elements of CATS have been discussed as mediating factors between social position and health outcomes capable of initiating spirals of behavioural mal-adaption and pathophysiological changes [65,66]. A cross-sectional analysis [86] corroborated the explanatory value of CATS for health inequalities, revealing:

- social gradients in the three kinds of outcome expectancies (positive, no control and negative) in the expected directions, and
- positive correlations between a positive outcome expectancy and active, instrumental coping strategies, as well as between generalised no control- and negative outcome expectancies and passivity and depressiveness (see also [87]).

Likewise, social networks (condition resource in Hobfoll’s COR theory) may add up to the psychosocial reserve aggregate in the reserve capacity model. The MOVE field study demonstrated that residents exposed to higher levels of air and noise pollution had a less dense social network relevant for both perceived behavioural control and performed institutional coping [27] (see the last paragraph in Section 3.2).

The reserve capacity model is primarily concerned with social stressors (exposures). Nonetheless, we link the moderation assumption to another line of argumentation: The attenuating effect of psychosocial resources on the association between environmental exposures like air pollution and (subclinical) physical health outcomes has been conceptualised in frameworks of environmental justice [5,22,88,89,90]. Correspondingly, psychosocial resources including generalised stimuli and outcome expectancies of the reserve capacity can be considered as effect modifiers in the direct pathway between personal traffic-noise exposure and health, as they confer psychological vulnerability and physiological susceptibility to exposures (incl. the feedback from the reserve capacity to stress due to perceived or feared resource loss).

Reserve capacities need time to build up, as do residents’ generalised and stimulus-specific expectancies of stress stimuli and outcomes (see Section 4.1). In the category of psychosocial resources, we should therefore consider previous experiences and individual conditions affecting current cognitive-motivational processes (condition resources). If perceived as effective, previous civic engagement may encourage residents to expect more control over their traffic-related exposure indoors and to develop positive outcome expectancies of future participation in environmental (noise action) planning (as suggested by environmental psychological models and studies on noise annoyance, see Section 3.1). Residents with chronic health conditions may see themselves confronted with repeated non-contingencies between compliance and health outcomes and may generalise this helplessness experience (cf. [48] for an example of learned helplessness induced by chronic pain (p. 146) or reflected by depression (pp. 250–251)). In a similar vein, we suggest that hearing impairments may render residents more prone to cognitive-motivational deficits and favour the generalisation of no control expectancies, as speech intelligibility and communication is further impeded by traffic-related sound masking [52,71]. To trace the spill-over effects of outcome expectancies in different stages in the life-course, we ought to include response-outcome-relations learned in different contexts (e.g., strained adaptive capacities due to high workloads and high social demands among middle-aged residents as indicated by higher vulnerability to noise annoyance [91]) and their cumulative (or even multiplicative) health effects [92].

**Figure 2:**
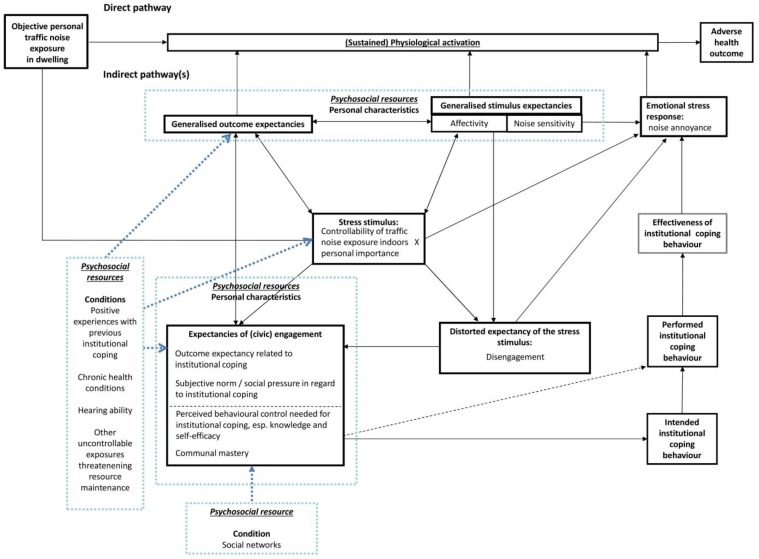
Adding psychosocial resources to our conceptual model.

Figure 2 exemplifies the potential “entry points” for psychosocial condition resources, in addition to personal characteristics.

### 5.2. Tangible Resources

In addition to the psychosocial resource aggregate, the reserve capacity model includes tangible resources, which remain somewhat elusive in the current state of this model. Tangible resources may be related to “financial and material goods, which might otherwise offset tangible stressors such as job loss, illness, or disability” (e.g., [32] p. 35). We suggest filling the tangible resource aggregate with objects (e.g., sound insulating windows), other conditions (e.g., time spent at home or being away), and energy resources (e.g., internet access). These objects and condition resources are studied as modifiers of the health effect of traffic noise [93]. Aside from moderating the objective exposure indoors [93,94], they may also contribute to maintaining or generating the psychosocial resource aggregate. For example, home ownership, recognised as a resource for behavioural control and institutional coping behaviour in MOVE, and a secondary residence, may convey a sense of having opportunities and being in control of one’s living environment(s), thereby tempering the perceived constraints that a resident may associate with escaping from traffic noise exposure (see [95] for a similar argumentation). Further, internet access has turned out to be an important source of environmental and political knowledge. Obliged to provide free access to environmental information by European legislation, municipalities increasingly use the internet as a dissemination tool. In fact, e-participation has been tested in a pilot study in the context of noise action planning [18] (see Section 1).

Finally, we may use some aspects of the soundscape approach [96] to make explicit tangible condition resources in the residential environment where residents make stressful or resource-replenishing experiences [31,32,85]. Traffic loads in the residential environment are linked to urban functions and road networks. Built and green environments may buffer or mask traffic noise levels and strengthen the perceived control over noise exposure via physical abatement, audio-visual shielding, or natural soundscapes (see the study by [93] with respect to the association between quiet sides and annoyance due to road traffic, further empirical studies [97,98,99] and reviews [100,101,102]). What is more, if we focus on resources as required by MOVE, we should shed light on the relationships between the stimulus and outcome expectancies in the absence of personal noise exposure: “According to the conservation of resource model, when not currently confronted with stressors, people strive to develop resource surpluses in order to offset the possibility of future resource loss” ([38] p. 517). In this way, we may improve our understanding of whether or to what extent cognitive-motivational resources are rooted in a residential environment free of the ambient stressor traffic noise.

**Figure 3:**
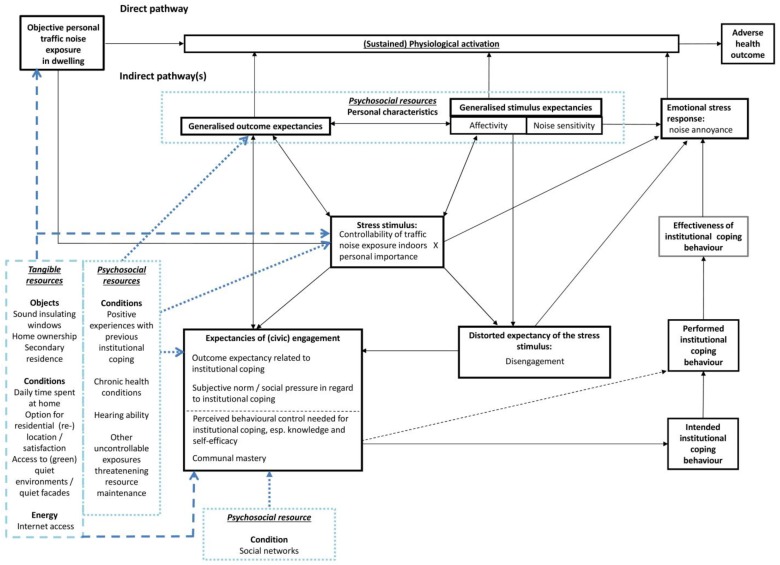
Adding tangible resources to our conceptual model.

Figure 3 shows the potential “entry points” for tangible resources.

### 5.3. Social Position

The income (energy resource) and education, age, and gender (condition resources) are constituents of residents’ social position. The different conditions and energies of social position can enhance or diminish differential environmental exposures and facilitate or impair differential access to resources (re-)produced and (re-)allocated by the different logics of market, welfare, and civil society [103,104]. To fathom health inequities, key relations hypothesised in our refined conceptual model should be tested in subgroups defined by energy and condition resources (see the study by [105], for example). If learning response-outcome expectancies is a key driver of loss or gain cycles, it is reasonable to assume that trajectories of mental and physical health are interconnected with social positioning over the life-course, as postulated by the reserve capacity model.

## 6. Intervention Opportunities Based on Our Conceptual Model

If confirmed in empirical analyses, our model may justify intervention opportunities regarding:

- environmental and planning determinants (e.g., direct contributions to resource aggregates by means of distributional “amendments” in the soundscape of residential environment, see the project “QUADMAP QUiet Areas Definition & Management in Action Plans” [106], and inplanning procedures promoting positive behaviour-outcome relationships);
- stimulus characterisations (e.g., figuring out where noise attributions are objectively unrealistic or wrong);
- effective response options (e.g., learning new skills, expanding the coping repertoire, and rectifying expectations of outcomes during participation processes).

for example (see [47] for entry points for intervention in the original reformulated learned helplessness model as well as [107,108] pp. 364–365 for the idea of “empowerment through control” among others authors). In doing so, planners should be aware of their own constraints and opportunities before mobilising attention for participation in noise action planning and raising residents’ expectations, given that “[…] control beliefs can be associated with poorer health outcomes under certain circumstances, especially when expectations for control are high but opportunities to exercise it are constrained. […]” ([87] p. 211).

More specifically, new noise priority indices could be developed using vulnerability profiles according to the four patterns of cognitive-motivational determinants. As illustrated by the so-called HARMONICA index for a better comprehensibility of information on environmental noise exposure [13], results from public participation could be useful for the validation of such an index. When designing participatory schemes, the patterns displayed in Table 1 may help include those who are likely to be excluded and those who are likely to be critical of noise action planning. Finally, the patterns may inform schemes for evaluating the impact of noise abatement measures on social inequalities in exposure to traffic-related noise and health outcomes.

## 7. Conclusions

In view of increasing traffic loads [109,110,111], the END is supposed to protect and improve residents’ health through noise action plans in urban areas. This paper raised concerns that a sectoral, population-based intervention like noise action planning might account for additional health inequities via (unintended) procedural injustice, as socially unequal participation may produce further disproportionate exposures ([69], see [112] for a similar argumentation in this context).

Due to its resource perspective, MOVE offers a compelling theoretical basis to examine social differences in civic engagement for environmental quality. Environmental psychological evidence points out the adverse effects of intractable and chronic traffic noise on cognitions and emotions. Using CATS and the reserve capacity model, we outlined a new conceptual model elucidating the linkages between cognitive-motivational determinants of residents’ civic engagement and health (inequities) for the first time. By describing cognitive-motivational vulnerability profiles, this model stresses the need to target socially different population groups instead of focusing on the population average, as is mostly frequently done in current environmental planning practice.

If validated in empirical studies, our conceptual model may indicate how to transform noise action planning into an instrument conducive to health equity and could serve as a logic basis for evaluation.

### Notes

(1) At this point, it would be worthwhile examining health differentials in relation to different coping strategies in the context of chronic noise exposure, as done by van Kamp [57]. This study used the Lazarus appraisal model on stress, coping, and health, while integrating the CATS notion of physiological activation. The Lazarus model explains the stress process as it evolves from a specific source of stress and specific coping strategies. Conceptual parallels were drawn between the Lazarus’ primary appraisal and stimulus expectancy, as well as secondary appraisal and outcome expectancy [57]. However, CATS theorises the stress process on a more general level by conceptualizing expectancies as decisive stress moderators and coping is defined as a positive outcome expectancy of any behavioural response that an individual selects to face stress stimuli. Interestingly, the authors of CATS deduced this coping concept from an experimental finding: “It was not the performance, or the feedback from evaluation of the performance, that mattered, it was the subjective feeling of being able to perform that reduced the stress responses” ([30] p. 878). This conception fits the TPB predictors of coping intentions and coping performance. Moreover, CATS outcome expectancies were shown to be translated into actual coping strategies, motivational and emotional responses ([86], see Section 5.1). However, results on the beneficial health effects of problem-focused coping were somewhat ambivalent [57]. There was no correlation between problem-focused coping and subjective health complaints. Residents having a pronounced inclination to apply problem-focused coping fared better in terms of subjective health when higher levels of noise stress were present. At the same time, this type of strategy yielded higher levels of blood pressure, which might indicate a latent, still unconscious health-related loss cycle due to an imbalance between resource investment and replenishment. Similarly, a recent analysis indicated a protective effect of problem-focused coping (reducing noise exposure indoors by closing windows, wearing earplugs; seeking contact with neighbours and municipality) on subjective health [113]. Physiological measures were lacking in this study.

(2) The current literature suggests dependencies between negative affectivity, noise sensitivity, noise annoyance, and (subjective) health. The relationship between negative affectivity and noise sensitivity still remains controversial [114].

(3) The authors of the reserve capacity model stress that the evidence base for the reserve capacity model is far from being consistent and complete, especially with respect to the tenet of increased stress reactivity in socially disadvantaged groups. In addition, the authors point out that empirical evidence rests on studies treating resources as separate factors, regardless of the tendency of resources to accumulate as claimed by COR theory. There is little evidence on resource aggregates, mostly provided by the authors themselves and confirming the mediational part. In doing so, they constructed composite variables aggregating psychosocial resources. However, the interactive and synergistic effects of resources and the relative contribution of each resource remain largely unexplored—in spite of their relevance for interventions in our view. We believe that the modelling of the “aggregate” needs more exploration, in the form of cross-sectional and longitudinal analyses.

## Figures and Tables

**Table 1:** Hypothetical patterns of cognitive-motivational determinants, noise annoyance, and sustained physiological activation within four groups.

Determinant	High Controllability x Low Personal Importance (Group 1)		High Controllability x High Personal Importance (Group 2)	Low Controllability x Low Personal Importance (Group 3)	Low Controllability x High Personal Importance (Group 4)	
noise annoyance	low		moderate	moderate	high	
noise sensitivity	low		high	low	high	
	A	B	-	-	A	B
generalised outcome expectancy	positive	no control	positive	no control	negative	positive
negative affect	low	high	low	high	high	low
positive affect	high	low	high	low	low	high
positive expectancies of institutional coping (civic engagement)	moderate	low	high	low	low	high
disengagement (cognitive distortion)	moderate	high	low	high	high	low
physiological activation ^1^	low	moderate	moderate	moderate	high	moderate

^1^ regardless of objective noise exposure levels.

## References

[1] WHO Regional Office for Europe (2011). Burden of Disease from Environmental Noise. Quantification of Healthy Life Years Lost in Europe.

[2] DIRECTIVE 2002/49/EC Relating to the Assessment and Management of Environmental Noise [Internet]. http://eur-lex.europa.eu/LexUriServ/LexUriServ.do?uri=OJ:L:2002:189:0012:0025:EN:PDF.

[3] Rose G. (1985). Sick individuals and sick populations. Int. J. Epidemiol..

[4] Kruize H. (2007). On environmental equity. Exploring the Distribution of Environmental Quality among Socio-Economic Categories in The Netherlands; Netherlands Geographical Studies 359.

[5] Bolte G., Pauli A., Hornberg C., Nriagu J.O. (2011). Environmental justice—Social disparities in environmental exposures and health. Overview. Encyclopedia of environmental health.

[6] Riedel N., Hoffmann B., Fuks K., Jöckel K.-H., Dragano N. (2011). Health inequalities in the city: Assessing the concurrence of social and environmental risks in the Ruhr Area. UMID.

[7] Bolte G., Bunge C., Hornberg C., Köckler H., Mielck A., Bolte G., Bunge C., Hornberg C., Köckler H., Mielck A. (2012). Umweltgerechtigkeit durch Chancengleichheit bei Umwelt und Gesundheit. Eine Einführung in die Thematik und Zielsetzung dieses Buches. Umweltgerechtigkeit. Chancengleichheit bei Umwelt und Gesundheit: Konzepte, Datenlage und Handlungsperspektiven.

[8] D’Alessandro F., Schiavoni S. (2015). A review and comparative analysis of European priority indices for noise action plans. Sci. Total Environ..

[9] Steinbach R., Grundy C., Edwards P., Wilkinson P., Green J. (2011). The impact of 20 mph traffic speed zones on inequalities in road casualties in London. J. Epidemiol. Community Health.

[10] Kruize H., Bolte G., Bunge C., Hornberg C., Köckler H., Mielck A. (2012). Einfluss eines neuen Verkehrsplans (VCP) auf Personen mit unterschiedlich hohem Einkommen: Erfahrungen aus der Stadt Den Haag in den Niederlanden. Umweltgerechtigkeit. Chancengleichheit bei Umwelt und Gesundheit: Konzepte, Datenlage und Handlungsperspektiven.

[11] Tonne C., Beevers S., Armstrong B., Kelly F., Wilkinson P. (2008). Air pollution and mortality benefits of the London Congestion Charge: Spatial and socioeconomic inequalities. Occup. Environ. Med..

[12] Schulze-Fielitz H. (2009). Brauchen wir eine Verordnung zur Lärmaktionsplanung?. NuR.

[13] Mietlicki F., Mietlicki C., Ribeiro C., Gaudibert P., Vincent B., Gissinger B. www.noiseineu.eu: New Tools to Inform the Public about Environmental Noise in Cities and to Assist Decision-Making. Proceedings of the Euronoise Conference.

[14] Schiewe J., Weninger B., Kornfeld A.L. (2012). Forschungsprojekt OptiLAP—Evaluierung und Optimierung der Lärmaktionsplanung nach der Umgebungslärmrichtlinie 2002/49/EG. Arbeitspaket 2.1 Analyse und Verbesserung der Gebrauchstauglichkeit von Lärmkarten in der Öffentlichkeitsbeteiligung.

[15] Luzzi S., Bellomini R., Natale R., Bartalucci C., Borchi F., Carfagni M., Governi L. Importance of public participation in END implementation: Some experiences from Italian agglomerates and infrastructures. Proceedings of the Euronoise Conference.

[16] Supplies T., Elsässer R., Mothes F. (2013). Lärmminderung durch Bürgerbeteiligung Das Modellprojekt: Mach's—Mitwirken bei der Lärmaktionsplanung in Leipzig.

[17] Heinrichs E., Kumsteller F., Rath S., Seidel P., Gurok S. (2015). Lärmbilanz 2015. Wissenschaftlich-Technische Unterstützung bei der Datenberichterstattung zur Lärmaktionsplanung.

[18] Märker O., Basedow S., Wessel M., Lindloff C., Kuhlmann W., Cremer N. (2010). Elektronische Partizipation zur Lärmaktionsplanung in Essen. Essen-soll-Leiser-Werden.de.

[19] Köckler H. (2014). Nur die Einladung reicht nicht. Teilhabe als Schlüssel umweltbezogener Gerechtigkeit. Politische Ökol..

[20] Lercher P., Pfeiffer C., Botteldooren D., Dekoninck L. Traffic noise exposure, education, and annoyance: Longitudinal experiences from cross-sectional studies over time (1989–2004). Proceedings of the Forum Acusticum.

[21] McLaren L., McIntyre L., Kirkpatrick S. (2010). Rose’s population strategy of prevention need not increase social inequalities in health. Int. J. Epidemiol..

[22] De Fur P.L., Evans G.W., Cohen Hubal E.A., Kyle A.D., Morello-Frosch R.A., Williams D.R. (2007). Vulnerability as a function of individual and group resources in cumulative risk assessment. EHP.

[23] Köckler H., Hornberg C., Bolte G., Bunge C., Hornberg C., Köckler H., Mielck A. (2012). Vulnerabilität als Erklärungsmodell einer sozial differenzierten Debatte um Risiken und Chancen im Kontext von Umweltgerechtigkeit. Umweltgerechtigkeit. Chancengleichheit bei Umwelt und Gesundheit: Konzepte, Datenlage und Handlungsperspektiven.

[24] Kruize H., Droomers M., van Kamp I., Ruijsbroek A. (2014). What causes environmental inequalities and related health effects? An analysis of evolving concepts. Int. J. Environ. Res. Public Health.

[25] Baxter S., Killoran A., Kelly M.P., Goyder E. (2010). Synthesizing diverse evidence: The use of primary qualitative data analysis methods and logic models in public health reviews. Public Health.

[26] Anderson L.M., Petticrew M., Rehfuess E., Armstrong R., Ueffing E., Baker P., Francis D., Tugwell P. (2011). Using logic models to capture complexity in systematic reviews. Res. Synth. Methods.

[27] Köckler H. Vulnerabilität von Haushalten Gegenüber Ihrer Lokalen Umweltgute. Eine Analyse aus Planerischer Perspektive vor dem Hintergrund Umweltbezogener Gerechtigkeit.

[28] Köckler H. (2011). MOVE: Ein Modell zur Analyse umweltbezogener Verfahrensgerechtigkeit. Umweltpsychologie.

[29] Ursin H., Eriksen H.R. (2004). The cognitive activation theory of stress. Psychoneuroendocrinology.

[30] Ursin H., Eriksen H.R. (2010). Cognitive activation theory of stress (CATS). Neurosci. Biobehav. Rev..

[31] Matthews K.A., Gallo L.C., Taylor S.E. (2010). Are psychosocial factors mediators of socioeconomic status and health connections? A progress report and blueprint for the future. Ann. N. Y. Acad. Sci..

[32] Gallo L.C., Matthews K.A. (2003). Understanding the association between socioeconomic status and physical health. Do negative emotions play a role?. Psychol. Bull..

[33] Gallo L.C. (2009). The reserve capacity model as a framework for understanding psychosocial factors in health disparities. Appl. Psychol. Health Well-Being.

[34] Ajzen I. (1991). The theory of planned behavior. Organ. Behav. Hum. Decis. Process..

[35] Ajzen I. (2002). Perceived behavioral control, self-efficacy, locus of control, and the theory of planned behavior1. J. Appl. Soc. Pyschol..

[36] Ajzen I., Lange P.A.M., Kruglanski A.W., Higgins E.T. (2012). The theory of planned behavior. Handbook of Theories of Social Psychology.

[37] Ajzen I. The Theory of Planned Behavior. http://people.umass.edu/aizen/tpb.diag.html#null-link.

[38] Hobfoll S.E. (1989). Conservation of Resources. A New Attempt at Conceptualizing Stress. Am. Psychol..

[39] Hobfoll S.E., Jackson A.P. (1991). Conservation of resources in community intervention. Am. J. Community Psychol..

[40] Hobfoll S.E., Jackson A.P., Hobfoll I., Pierce C., Young S. (2002). The impact of communal-mastery versus self-mastery on emotional outcomes during stressful conditions: A prospective study of native American women. Am. J. Community Psychol..

[41] Hiroto D.S. (1974). Locus of control and learned helplessness. J. Exp. Psychol..

[42] Job R.F.S. (1996). The influence of subjective reactions to noise on health effects the noise. Environ. Int..

[43] Evans G.W., Baum A., Revenson T.A., Singer J.E. (2001). Environmental stress and health. Handbook of Health Psychology.

[44] Evans G.W., Cohen S., Spielberger C.D. (2004). Environmental stress. Encyclopedia of Applied Psychology.

[45] Evans G.W., Stecker R. (2004). Motivational consequences of environmental stress. J. Environ. Psychol..

[46] Hiroto D.S., Seligman M.E. (1975). Generality of learned helplessness in man. J. Personal. Soc. Psychol..

[47] Abramson L.Y., Seligman M.E., Teasdale J.D. (1978). Learned helplessness in humans. Critique and reformulation. J. Abnorm. Psychol..

[48] Peterson C., Maier S.F., Seligman M.E. (1993). Learned Helplessness. A Theory for the Age of Personal Control.

[49] Peterson C., Park C., Barone D.F., Hersen M., van Hasselt V.B. (1998). Learned helplessness and explanatory style. Advanced Personality.

[50] Peterson P., Park N., Gross J.J. (2007). Explanatory style and emotion regulation. Handbook of Emotion Regulation.

[51] Campbell J.M. (1983). Ambient stressors. Environ. Behav..

[52] WHO Regional Office for Europe (1999). Guidelines for Community Noise.

[53] Flade A., Schöller O., Canzler W., Knie A. (2007). Die sozialen Kosten des Verkehrs. Handbuch Verkehrspolitik.

[54] Claßen T. (2013). Lärm macht krank—Gesundheitliche Wirkungen von Lärmbelastungen in den Städten. IzR.

[55] Stallen P.J.M. (1999). A theoretical framework for environmental noise annoyance. Noise Health.

[56] Klæboe R., Nriagu J.O. (2011). Noise and health—Annoyance and Interference. Encyclopedia of Environmental Health.

[57] Van Kamp I. (1990). Coping with Noise and Its Health Consequences.

[58] Hatfield J., Job R., Soames F., Hede A.J., Carter N.L., Peploe P., Taylor R., Morrell S. (2002). Human response to environmental noise: The role of perceived control. IJBM.

[59] Kroesen M., Molin E.J.E., van Wee B. (2008). Testing a theory of aircraft noise annoyance. A structural equation analysis. JASA.

[60] Schreckenberg D., Meis M., Kahl C., Peschel C., Eikmann T. (2010). Aircraft noise and quality of life around Frankfurt airport. Int. J. Environ. Res. Public Health.

[61] Guski R. (1999). Personal and social variables as co-determinants of noise annoyance. Noise Health.

[62] Flindell I.H., Stallen P.J.M. (1999). Non-acoustical factors in environmental noise. Noise Health.

[63] Kroesen M., Molin E.J.E., van Wee B. (2010). Determining the direction of causality between psychological factors and aircraft noise annoyance. Noise Health.

[64] Eriksen H.R., Ursin H. (2002). Social inequalities in health: biological, cognitive and learning theory perspectives. Nor. Epidemiol..

[65] Kristenson M., Eriksen H.R., Sluiter J.K., Starke D., Ursin H. (2004). Psychobiological mechanisms of socioeconomic differences in health. Soc. Sci. Med..

[66] Ree E., Odeen M., Eriksen H.R., Indahl A., Ihlebaek C., Hetland J., Harris A. (2014). Subjective health complaints and self-rated health: Are expectancies more important than socioeconomic status and workload?. IJBM.

[67] McEwen B.S. (1998). Stress, Adaptation, and disease. allostasis and allostatic load. Ann. N. Y. Acad. Sci..

[68] Von Lindern E., Hartig T., Lercher P. (2016). Traffic-related exposures, constrained restoration, and health in the residential context. Health Place.

[69] Freudenberg N., Pastor M., Israel B. (2011). Strengthening community capacity to participate in making decisions to reduce disproportionate environmental exposures. Am. J. Public Health.

[70] Smith A. (2013). The concept of noise sensitivity: Implications for noise control. Noise Health.

[71] Miedema H.M.E. (2007). Annoyance caused by environmental noise: Elements for evidence-based noise policies. J. Soc. Issues.

[72] Schreckenberg D., Griefahn B., Meis M. (2010). The associations between noise sensitivity, reported physical and mental health, perceived environmental quality, and noise annoyance. Noise Health.

[73] Leganger A., Kraft P., RØysamb E. (2000). Perceived self-efficacy in health behaviour research. Conceptualisation, measurement and correlates. Psychol. Health.

[74] Steptoe A., Dockray S., Wardle J. (2009). Positive affect and psychobiological processes relevant to health. J. Pers..

[75] Ward M.M. (2013). Sense of control and self-reported health in a population-based sample of older Americans: Assessment of potential confounding by affect, personality, and social support. IJBM.

[76] Basner M., Babisch W., Davis A., Brink M., Clark C., Janssen S., Stansfeld S. (2014). Auditory and non-auditory effects of noise on health. Lancet.

[77] Recio A., Linares C., Banegas J.R., Diaz J. (2016). Road traffic noise effects on cardiovascular, respiratory, and metabolic health: An integrative model of biological mechanisms. Environ. Res..

[78] Babisch W. (2002). The noise/stress concept, risk assessment and research needs. Noise Health.

[79] Babisch W., Pershagen G., Selander J., Houthuijs D., Breugelmans O., Cadum E., Vigna-Taglianti F., Katsouyanni K., Haralabidis A.S., Dimakopoulou K. (2013). Noise annoyance—A modifier of the association between noise level and cardiovascular health?. Sci. Total Environ..

[80] Brunner E. (1997). Socioeconomic determinants of health: Stress and the biology of inequality. BMJ.

[81] Brunner E. (2007). Biology and health inequality. PLoS Biol..

[82] Brunner E., Marmot M., Marmot M., Wilkinson R.G. (2006). Social organization, stress, and health. Social Determinants of Health.

[83] McEwen B.S., Gianaros P.J. (2010). Central role of the brain in stress and adaption: Links to socio-economic status, health, and disease. Ann. N. Y. Acad. Sci..

[84] Krieger N. (2011). Epidemiology and the People's Health. Theory and Context.

[85] Daniel M., Moore S., Kestens Y. (2008). Framing the biosocial pathways underlying associations between place and cardiometabolic disease. Health Place.

[86] Odeen M., Westerlund H., Theorell T., Leineweber C., Eriksen H.R., Ursin H. (2013). Expectancies, socioeconomic status, and self-rated health: Use of the simplified TOMCATS Questionnaire. IJBM.

[87] Taylor S.E., Seeman T.E. (1999). Psychosocial resources and the SES-health relationship. Ann. N. Y. Acad. Sci..

[88] Gee G.C., Payne-Sturges D.C. (2004). Environmental health disparities: A framework integrating psychosocial and environmental concepts. EHP.

[89] Morello-Frosch R., Shenassa E.D. (2006). The environmental “riskscape” and social inequality: Implications for explaining maternal and child health disparities. EHP.

[90] McEwen B.S., Tucker P. (2011). Chemical biological pathways for chronic psychosocial stress and research opportunities to advance the consideration of stress in chemical risk assessment. Am. J. Public Health.

[91] Van Gerven P.W.M., Vos H., van Boxtel M.P.J., Janssen S.A., Miedema H.M.E. (2009). Annoyance from environmental noise across the lifespan. JASA.

[92] Riedel N., Loerbroks A., Bolte G., Li J. (2017). Do perceived job insecurity and annoyance due to air and noise pollution predict self-rated poor health? A prospective analysis of independent and joint associations using a German national representative cohort study. BMJ Open.

[93] Babisch W., Swart W., Houthuijs D., Selander J., Bluhm G., Pershagen G., Dimakopoulou K., Haralabidis A.S., Katsouyanni K., Davou E. (2012). Exposure modifiers of the relationships of transportation noise with high blood pressure and noise annoyance. JASA.

[94] Foraster M., Künzli N., Aguilera I., Rivera M., Agis D., Vila J., Bouso L., Deltell A., Marrugat J., Ramos R. (2014). High blood pressure and long-term exposure to indoor noise and air pollution from road traffic. EHP.

[95] Forastiere F., Stafoggia M., Tasco C., Picciotto S., Agabiti N., Cesaroni G., Perucci C.A. (2007). Socioeconomic status, particulate air pollution, and daily mortality: Differential exposure or differential susceptibility. Am. J. Ind. Med..

[96] Kang J., Schulte-Fortkamp B. (2016). Soundscape and the Built Environment.

[97] de Kluizenaar Y., Salomons E.M., Janssen S.A., van Lenthe F.J., Vos H., Zhou H., Miedema H.M.E., Mackenbach J.P. (2011). Urban road traffic noise and annoyance: The effect of a quiet façade. JASA.

[98] Gidlöf-Gunnarsson A., Öhrström E. (2007). Noise and well-being in urban residential environments: The potential role of perceived availability to nearby green areas. Landsc. Urban Plan..

[99] Gidlöf-Gunnarsson A., Öhrström E. (2010). Attractive “quiet” courtyards: A potential modifier of urban residents’ responses to road traffic noise?. Int. J. Environ. Res. Public Health.

[100] Dzhambov A.M., Dimitrova D.D. (2014). Urban green spaces’ effectiveness as a psychological buffer for the negative health impact of noise pollution: A systematic review. Noise Health.

[101] Claßen T., Jäcker-Cüppers M., Riedel N., Kowarik I., Bartz R., Brenck M. (2016). Weniger Lärm. Ökosystemleistungen in der Stadt Gesundheit schützen und Lebensqualität erhöhen. Stadtbericht. Naturkapital Deutschland—TEEB DE.

[102] Van Kamp I., Klaeboe R., Brown A.L., Lercher P., Kang J., Schulte-Fortkamp B. (2016). Soundscapes, human restoration and quality of life. Soundscape and the Built Environment.

[103] Musterd S., Murie A., Kesteloot C. (2006). Neighbourhoods of Poverty. Urban Social Exclusion and Integration in Comparison.

[104] Bernard P., Charafeddine R., Frohlich K.L., Daniel M., Kestens Y., Potvin L. (2007). Health inequalities and place: A theoretical conception of neighbourhood. Soc. Sci. Med..

[105] Riedel N., Fuks K., Hoffmann B., Weyers S., Siegrist J., Erbel R., Viehmann A., Stang A., Scheiner J., Dragano N. (2012). Insomnia and urban neighbourhood contexts—Are associations modified by individual social characteristics and change of residence? Results from a population-based study using residential histories. BMC Public Health.

[106] QUADMAP QUiet Areas Definition and Management in Action Plans Project Group Guidelines for the Identification, Selection, Analysis, and Management of Quiet Urban Areas, Update Version 2.0 March 2015. http://www.quadmap.eu/wp-content/uploads/2012/01/Guidelines_QUADMAP_ver2.0.pdf.

[107] Zimmerman M.A. (1990). Toward a theory of learned hopefulness: A structural model analysis of participation and empowerment. J. Res. Pers..

[108] Lercher P., Luxon L.M., Deepak P. (2007). Environmental noise: A contextual health perspective. Noise and its Effects.

[109] Umweltbundesamt Verkehr in Zahlen 2012. http://www.umweltbundesamt.de/sites/default/files/medien/publikation/long/4364.pdf.

[110] European Commission White Paper 2011. Roadmap to a Single European Transport Area—Towards a Competitive and Resource Efficient Transport System. http://eur-lex.europa.eu/legal-content/EN/TXT/PDF/?uri=CELEX:52011DC0144&from=EN.

[111] European Commission EU Transport in Figures. Statistical Pocketbook 2016. https://ec.europa.eu/transport/facts-fundings/statistics/pocketbook-2016_en.

[112] Riedel N., Köckler H., Scheiner J., Berger K. (2015). Residential road traffic exposure, noise annoyance, and self-rated poor health—A proposal for an analytical concept framing the relationship between noise and health as a matter of multiple stressors and resources in urban neighbourhoods. J. Environ. Plan. Manag..

[113] Lercher P., van Kamp I., von Lindern E. Transportation noise and health-related quality of life: Perceptions of soundscapes, coping, and restoration. Proceedings of the EuroNoise 2015.

[114] Shepherd D., Heinonen-Guzejev M., Heikkila K.M., Dirks K.N., Hautus M.J., Welch D., McBride D. (2015). The negative affect hypothesis of noise sensitivity. Int. J. Environ. Res. Public Health.

